# Outcomes of the novel Odon Device in indicated operative vaginal birth

**DOI:** 10.1016/j.ajog.2020.12.017

**Published:** 2021-06

**Authors:** Emily J. Hotton, Erik Lenguerrand, Mary Alvarez, Stephen O’Brien, Tim J. Draycott, Joanna F. Crofts, ASSIST Study Team, Mary Alvarez, Sabaratnam Arulkumaran, Nichola Bale, Natalie S. Blencowe, Joanna F. Crofts, Timothy J. Draycott, Lily Exell, Anne Glover, Sally Hall, Emily J. Hotton, Erik Lenguerrand, Helen Lewis-White, Naomi Mallinson, Michelle Mayer, Sadie McKeown-Keegan, Glen Mola, Stephen O’Brien, Alison Pike, Iona Smith, Claire Rose, Sherrie Villis, Julia Wade, Paul White, Cathy Winter

**Affiliations:** a Women and Children’s Research Centre, Southmead Hospital, Bristol, United Kingdom; b Translational Health Science, University of Bristol, Southmead Hospital, Bristol, United Kingdom; c Population Health Science, University of Bristol, Bristol, United Kingdom; d Maternity Unit, Royal United Hospitals Bath NHS Foundation Trust, Bath, United Kingdom

**Keywords:** assisted vaginal birth, feasibility, fetal compromise, intrapartum research, management of second stage of labor, medical device, nonreassuring fetal heart tracing, obstetrical forceps, prolonged second stage of labor, safety, vacuum, ventouse

## Abstract

**Background:**

No new method of assisting vaginal birth has been introduced into clinical practice since the development of the vacuum extractor in the 1950s. The Odon Device is a new device that employs a circumferential air cuff over the fetal head to assist birth. In this study, the Odon Device has been used to assist vaginal birth for standard clinical indications.

**Objective:**

This study aimed to investigate the clinical impact, safety, and acceptability of the Odon Device to women, their babies, and clinicians and to assess the feasibility of recruiting women to an interventional intrapartum research study.

**Study Design:**

This is a nonrandomized, single-arm interventional feasibility study of the Odon Device for operative vaginal birth undertaken in a single maternity unit: Southmead Hospital, Bristol, United Kingdom. The Odon Device was used to assist birth in 40 women who required the birth to be assisted for suspected fetal compromise and/or prolonged second stage of labor. The primary clinical outcome was the proportion of births successfully assisted with the Odon Device, and the primary feasibility outcome was the proportion of eligible women who were approached and who agreed to participate. Neonatal outcome data were reviewed at day 28, and maternal outcomes were investigated up to day 90.

**Results:**

Between October 2018 and January 2019, 298 of 384 approached, eligible women (77.6%) consented to participate. Of these women, 40 received the intervention—the use of the Odon Device. Birth was assisted in all cephalic (occiput anterior, occiput transverse, and occiput posterior) fetal positions, at all stations at or below the ischial spine and with or without regional analgesia. The Odon Device was effective in 19 of 40 cases (48%). Of the 40 births, 21 (52.5%) required additional assistance: 18 of 40 births (45%) were completed using nonrotational forceps, 1 of 40 births (3%) required rotational forceps, and 2 of 40 births (5%) required an emergency cesarean delivery. There was no serious maternal or neonatal adverse event related to the use of the device, and there was no serious adverse device effect. There were 4 devices (10%) that were ineffective because of a manufacturing fault. Furthermore, 39 of 40 women (98%) reported a high birth perception score. All practitioners were able to use the device as intended, although some steps in using the device were reported to be easier to perform (setup and deflation of air chamber) than others (application of the device and withdrawal of the applicator).

**Conclusion:**

Recruitment to an interventional study of a new device for operative vaginal birth was feasible; 78% of eligible women were willing to participate, often expressing an aspiration for an alternative to forceps and vacuum. The success rate of the Odon Device was lower than reported success rates of vacuum and forceps; however, in this study, the device had been used to assist birth for standard clinical indications. There was no significant maternal or neonatal safety concern associated with the use of the device, although the number of births studied was small. Further feasibility study to establish iterative changes to the device, technique, and clinical indications is necessary.

**AJOG at a Glance**

**Why was this study conducted?**

No new method of operative vaginal birth has been introduced since the development of the vacuum extractor in the 1950s. The Odon Device is a new type of instrument that uses a circumferential air cuff over the fetal head and offers an alternative to vacuum and forceps.

**Key findings**

There was no serious maternal or neonatal adverse event related to the use of the Odon Device. In the first 40 clinically indicated cases ever to be conducted, the Odon Device was successful in 19 (48%), and there was a high maternal birth perception score.

**What does this add to what is known?**

The Odon Device currently has a lower success rate than current devices, but this should improve with technique refinements. High recruitment rates to studies of novel devices to assist birth are feasible.

## Introduction

The optimal duration of the second stage of labor is contentious,^1^, ^2^, ^3^, ^4^ but complications in the second stage of labor remain a major cause of preventable maternal and neonatal morbidities and mortalities globally.^4^ Skilled operative vaginal birth improves the outcomes for women and their babies when birth is assisted in the second stage of labor for suspected fetal compromise and/or delay^4^^,^^5^ compared with cesarean delivery. Despite this fact, the rate of cesarean delivery is increasing, whereas the rate of operative vaginal birth is low or nonexistent in many health services (reported rates as low as 0.5% of births).^6^ The reasons for low operative vaginal birth rates include inexperienced healthcare workers, lack of equipment, and operator and patient dislike of currently available methods.^6^ A new device to assist vaginal birth provides an opportunity to improve outcomes and reinvigorate this essential life-saving skill. However, in contrast to the huge advances in medical care over the past 70 years, no new method for assisting vaginal birth has been introduced into clinical practice since the development of the vacuum extractor in the 1950s. The advantages of the Odon Device compared with the advantages of standard methods for assisting birth (ie, forceps and vacuum) are currently unknown as the device has not yet been used in clinically indicated cases. Potential advantages could include improved neonatal outcomes (eg, reduction in subaponeurotic and retinal hemorrhages, skull fracture, and facial nerve palsy), increased maternal acceptability, reduction in failed rate of operative vaginal birth, decreased risk of adverse maternal outcomes (eg, postpartum hemorrhage and vaginal tears), and ease of use (ie, the same application technique of the device for all cephalic positions).

**Figure 1:**
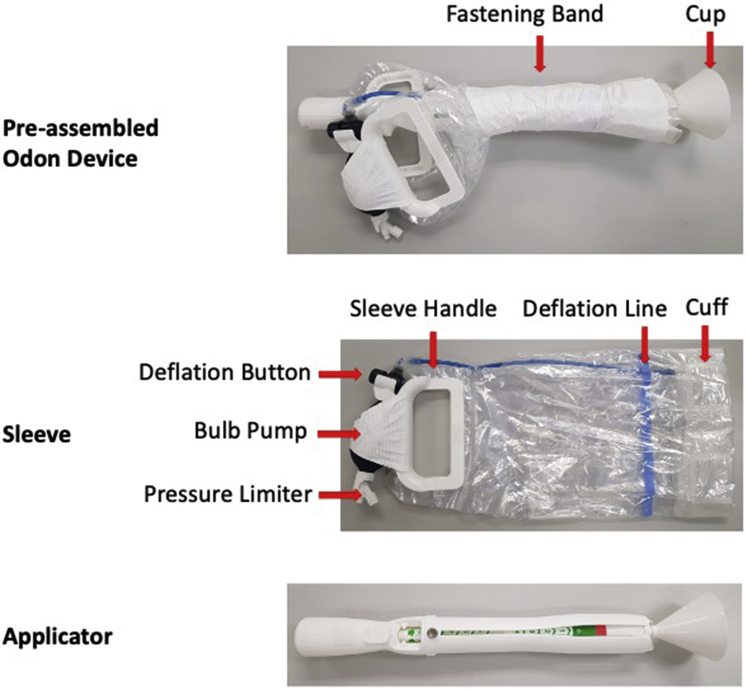
The Odon Device component parts *Hotton et al. Novel Odon Device and operative vaginal birth. Am J Obstet Gynecol 2021.*

**Supplemental Figure 1:**
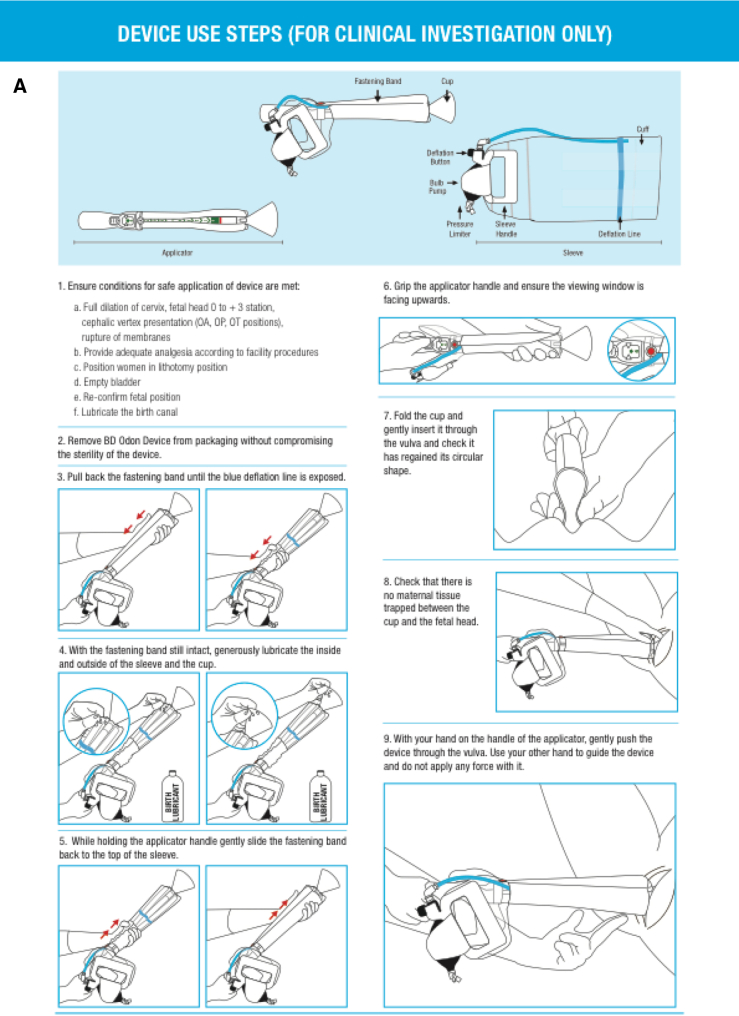

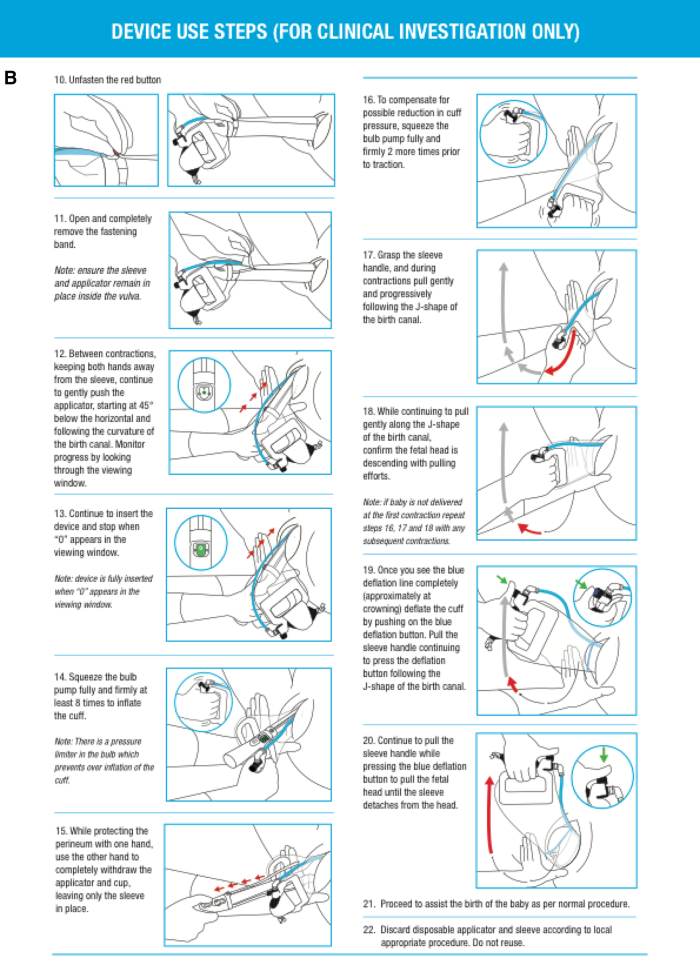
Instructions for use for the ASSIST Study **A and B,** Device use steps (for clinical investigation only). *Hotton et al.Novel Odon Device and operative vaginal birth. Am J Obstet Gynecol 2021*.

The Odon Device is a new device that can be used for operative vaginal birth (Figure 1) consisting of a plastic applicator and polyethylene sleeve. The applicator has 4 flexible spatulas that position the sleeve over the fetal head. A progress indicator allows the operator to confirm the correct depth of insertion. The sleeve contains a circumferential air chamber that is inflated around the fetal head, providing the grip for the operator to apply traction (Video). The instructions for the use of the Odon Device are shown in Supplemental Figure 1. A phase 1 study of 4 earlier versions of the Odon Device in 49 healthy volunteers (women who were about to have a spontaneous vaginal birth) demonstrated that the device could be applied to the fetal head during the second stage of labor with no serious safety concern.^7^ Animal studies were subsequently performed for safety, and an extensive program of simulation studies confirmed that the device could be reliably sited and used in occiput anterior, occiput transverse, and occiput posterior fetal positions.^10^, ^11^, ^8^, ^9^

The ASSIST Study used the Odon Device to assist birth for routine clinical indications: suspected fetal compromise and/or failure to progress in the second stage of labor. The study aimed to investigate the feasibility of recruitment and to investigate the efficacy, safety, and acceptability of the Odon Device to women and their babies, midwives, obstetricians, and neonatologists.

## Materials and Methods

### Study design

This is a nonrandomized, single-arm feasibility study of the Odon Device for 40 women who required an operative vaginal birth for a recognized clinical indication. Qualitative work that explored the experience and views of women and clinicians will be published in full, separately.

### Population

**Figure 2:**
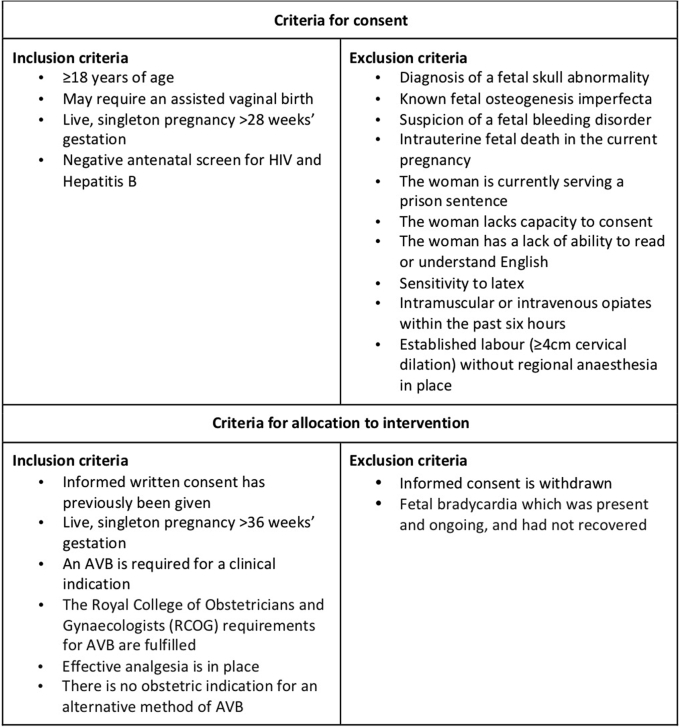
Consent and allocation to the intervention criteria *AVB*, atrioventricular block. *Hotton et al. Novel Odon Device and operative vaginal birth. Am J Obstet Gynecol 2021.*

Potential participants were approached at Southmead Hospital, Bristol, United Kingdom. Figure 2 outlines the eligibility criteria for initial consent and allocation to the intervention if an operative vaginal birth for a clinical indication was required for prolonged second stage of labor or presumed fetal compromise—as defined by the Royal College of Obstetricians & Gynaecologists.^12^ Women were provided with study information through a verbal discussion, an information leaflet, and a video demonstrating the Odon Device.

### Intervention

**Supplemental Figure 2:**
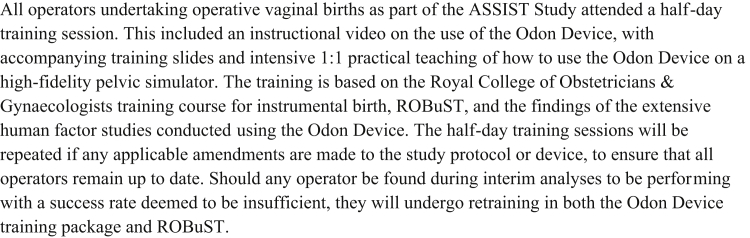
Operator training for the ASSIST Study *ROBuST*, RCOG Operative Birth Simulation Training. *Hotton et al.Novel Odon Device and operative vaginal birth. Am J Obstet Gynecol 2021*.

Odon Device–assisted births were conducted by 1 of 5 obstetricians who had undergone specialized training (Supplemental Figure 2). If the Odon Device was unsuccessful, the obstetrician used their clinical judgment to complete the birth using forceps, vacuum, or cesarean delivery. After use (following high-level disinfection), devices were systematically examined by the study team and manufacturer. The device was used in women who had a fully dilated cervix, with the vertex presenting at or below the ischial spines, in any position.

### Outcomes

The primary feasibility outcome was the proportion of approached and eligible women agreeing to participate. The primary clinical outcome was the proportion of births successfully assisted with the Odon Device. A birth was defined as “successful” if all of the following 6 criteria were met: (1) vaginal birth assisted with the Odon Device, (2) no serious maternal adverse reaction^13^ related to the use of the device, (3) no serious neonatal adverse reaction^13^ related to the use of the device, (4) no serious adverse device effect,^13^ (5) woman’s perception of her birth of >6 (Patient Perception Score [PPS]^14^) (Supplemental Table 1), and (6) practitioner-reported outcome of >12 (Supplemental Table 2).

**Figure 3:**
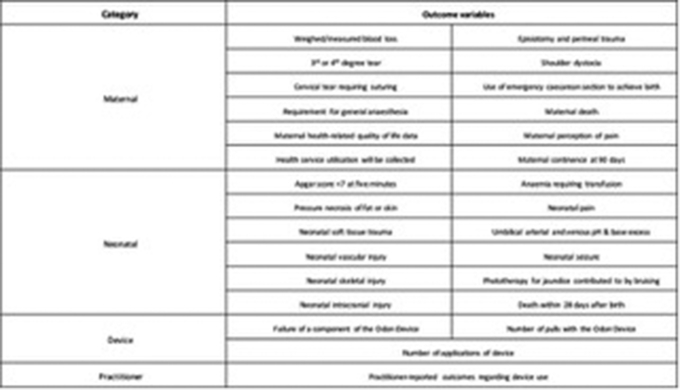
Secondary outcomes *BD*, Becton, Dickinson and Company. *Hotton et al. Novel Odon Device and operative vaginal birth. Am J Obstet Gynecol 2021.*

The PPS has been previously validated for operative vaginal birth.^14^ Operators were asked their perceptions of the use of the device using a simple, nonvalidated tool (Supplemental Table 2). Secondary outcomes included metrics related to study feasibility and safety (ie, maternal perineal trauma and measured blood loss, neonatal soft tissues trauma and pain, and device safety) (Figure 3). Neonates were followed up to day 28 via notes review and mothers on days 7, 28, and 90 after birth via telephone consultation. Further details are contained in the published study protocol.^15^

### Sample size

Sample sizes between 24 and 50 have been recommended for feasibility studies.^16^^,^^17^ A complete sample size of 40 women was fixed a priori to enable the estimation of a potential rate of successful operative vaginal birth of 80% to within a 95% confidence interval (CI) of ±12%. This sample size will demonstrate the use of a secondary instrument of 50% to within a 95% CI of ±15%.

### Statistical analysis

Data were entered and stored on a bespoke study database (GeneSYS) designed and managed by the Clinical Trial and Evaluation Unit, University of Bristol, Bristol, United Kingdom. Data were analyzed using Stata (version 15.1, StataCorp, College Station, TX). Continuous variables were reported as mean and standard deviation or median and interquartile range; categorical variables were reported as frequency and percentages. Relationships among characteristics that affect the success of the Odon Device were explored using nonparametric tests, the Fisher exact test for categorical variables, and the Mann-Whitney test for continuous variables.

### Ethics

This study was approved by South Central–Berkshire REC, United Kingdom, on September 3, 2018 (18/SC/0344), the Medicines and Healthcare Products Regulatory Agency on August 9, 2018, and the Health Research Authority on September 3, 2018.

## Results

### Recruitment

**Figure 4:**
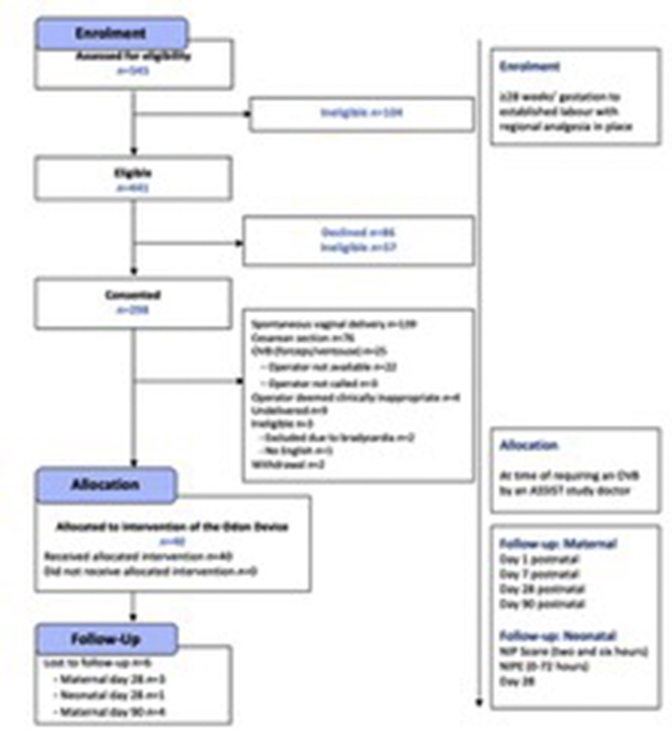
The ASSIST Study CONSORT diagram *AVB*, atrioventricular block; *CONSORT*, Consolidated Standards of Reporting Trials; *NIPE*, Newborn and Infant Physical Examination; *NIPS*, Neonatal Infant Pain Score. *Hotton et al. Novel Odon Device and operative vaginal birth. Am J Obstet Gynecol 2021.*

Women were recruited between October 2018 and January 2019 (Figure 4). Pregnancy notes of 545 women were screened; 441 of 545 women (80.9%) were initially deemed eligible and approached. Furthermore, 57 of the approached women were then identified to be ineligible. Of the 384 women who were approached and eligible, 298 (77.6%) consented to participate should they require an operative vaginal birth (Figure 4).

Of the 298 women who had consented, 224 (75.2%) did not have an operative vaginal birth: 139 (62.1%) had a spontaneous vaginal birth, 76 (33.9%) had a cesarean delivery, and 9 (4.0%) were not yet in labor when the recruitment target was reached. In addition, 72 women (24.2%) who had provided consent required an operative vaginal birth; the Odon Device was used in 40 cases (55.6%).

#### Data quality

There were minimal missing data: body mass index at 36 weeks’ gestation (6 of 40, 15%); umbilical artery pH and base excess (7 of 40, 18%); umbilical vein pH and base excess (4 of 40, 10%); Neonatal Infant Pain Score (NIPS)^18^ at 2 hours postnatal (1 of 40, 3%); and NIPS at 6 hours postnatal (5 of 40, 13%). Participants were deemed lost to follow-up if they did not respond within 4 days of the planned follow-up. Follow-up was not obtained from 3 (8%) and 4 (10%) women on day 28 and 90, respectively. A day 28 neonatal follow-up (3%) was not completed.

#### Demographics and characteristics

Table 1 summarizes the demographics and birth characteristics. Furthermore, 29 women (73%) had an operative vaginal birth for presumed fetal compromise and 11 women (28%) for prolonged second stage of labor. Birth was assisted in all cephalic fetal positions, at all stations at or below the ischial spines and with or without regional analgesia.

**Table 1:** Demographics and characteristics of participants

Variable	Overall (n=40)
Maternal age (y)	28.9±4.8
BMI at booking	25.3±5.5
BMI at 36 wk	29.4±5.7^a^
Ethnicity	
White British	32 (80.0)
Any other white background	4 (10.0)
Black African	1 (2.5)
Black Caribbean	1 (2.5)
Indian	1 (2.5)
Any other Asian background	1 (2.5)
Parity	
0	34 (85.0)
≥1	6 (15.0)
Previous cesarean delivery	1 (2.5)
Length of gestation (wk)	39.0±1.3
Birthweight (g)	3198±542 3120 (2800–3602)
Head circumference (cm)	34.0±1.6
Duration of first stage of labor (min)	342±199 321 (188–480)
Duration of second stage of labor (min)	112±63 103 (67–152)
Induced labor	34 (85.0)
Primary indication for operative vaginal birth	
Presumed fetal compromise	29 (72.5)
Prolonged second stage of labor	11 (27.5)
Fetal position	
OA	31 (77.5)
OT	4 (10.0)
OP	5 (12.5)
Fetal head palpable per abdomen	
0/5	40 (100.0)
1/5	0 (0.0)
Station of fetal head	
Spines	3 (7.5)
+1 cm below the spines	18 (45.0)
+2 cm below the spines	18 (45.0)
+3 cm below the spines	1 (2.5)
Molding	
None	9 (22.5)
+	26 (65.0)
++	4 (10.0)
+++	1 (2.5)
Caput	
None	11 (27.5)
1 cm	9 (22.5)
2 cm	16 (40.0)
3 cm	4 (10.0)
Analgesia	
None	1 (2.5)
Perineal infiltration	12 (30.0)
Pudendal block	1 (2.5)
Regional anesthesia	34 (85.0)
General anesthesia	0 (0.0)
Grade of operator	
Attending	29 (72.5)
Trainee	11 (27.5)

Data are presented as mean±standard deviation, number (percentage) or median (interquartile range).

*BMI*, body mass index; *OA*, occiput anterior; *OP*, occiput posterior; *OT*, occiput transverse.

*Hotton et al. Novel Odon Device and operative vaginal birth. Am J Obstet Gynecol 2021.*

a The number of participants is only 34.

#### Primary clinical outcome

The Odon Device was the only device required to assist birth in 19 of 40 cases (48%). There was no serious maternal or neonatal adverse reaction related to the use of the device during birth, and there was no serious adverse device effect. All practitioners found the device easy to use; however, 1 woman (3%) whose birth was assisted using the Odon Device reported her perception of the operative birth as poor. Therefore, by our a priori definition, the proportion of births that were “successfully” assisted with the Odon Device was 18 of 40 (45%).

Additional assistance was required to complete the birth in 21 of 40 cases (52%). Of the 40 cases, 18 (45%) were completed using nonrotational forceps, 1 (3%) required rotational forceps, and 2 (5%) required an emergency cesarean delivery (1 immediately following an unsuccessful Odon Device and 1 after a failed attempt using rotational forceps). The fetal vertex was at the level of the ischial spines in all cases requiring rotational forceps or cesarean delivery.

In addition, 39 of 40 devices (98%) were inspected after use, and 4 of 40 devices (10%) had a fault in the bulb pump mechanism, unable to inflate the air cuff. None of the faulty device had been successful, and in each case, the operator had raised concerns that the air chamber had not inflated adequately during use.

#### Characteristics that affect the success of the Odon Device

Table 2 summarizes the characteristics of 36 births, which were assisted with nonfaulty devices by success (19 cases) and failure (17 cases). Station (*P*=.014) and degree of molding (*P*=.022) were strongly related to the success of the device; higher fetal station and/or increased molding was associated with lower device success rates. Caput (*P*=.057) may also affect the success of the device; greater degrees of caput were associated with a higher likelihood of failure. There was no evidence to support any relationship among device success and onset of labor (*P*=.650), head circumference (*P*=.368), primary indication for operative vaginal birth (*P*=1.000), position of the fetal head (*P*=.843), birthweight (*P*=.272), length of the second stage of labor (*P*=.814), and length of the first stage of labor (*P*=.178).

**Table 2:** Demographics and characteristics for devices (excluding faulty devices)

	Successful Odon n=19	Unsuccessful Odon n=17
Maternal age (y)	28.10±5.40	29.90±4.20
BMI at booking	25.22±5.30	25.30±5.60
BMI at 36 wk	28.60±5.40^a^	29.60±6.00^b^
Ethnicity		
White British	15 (78.9)	15 (88.2)
Any other white background	3 (15.8)	0 (0.0)
Black African	0 (0.0)	1 (5.9)
Black Caribbean	0 (0.0)	0 (0.0)
Indian	1 (5.3)	0 (0.0)
Any other Asian background	0 (0.0)	1 (5.9)
Parity		
0	16 (84.2)	14 (82.3)
≥1	3 (15.8)	3 (17.6)
Previous cesarean delivery	0 (0.0)	1 (5.9)
Length of gestation (wk)	38.8±1.3	39.1±1.3
Birthweight (g)	3097±454 3044 (2730–3405)	3207±(606) 3190 (2724–3604)
Head circumference (cm)	34.00±1.50	34.00±1.50
Duration of first stage (min)	334±197 240 (180–485)	357±217 314 (240–480)
Duration of second stage (min)	97±54 100 (61–139)	122±64 122 (75–158)
Induced labor	17 (89.5)	14 (82.4)
Primary indication for operative vaginal birth		
Presumed fetal compromise	14 (73.7)	13 (76.5)
Delay in the second stage of labor	5 (26.3)	4 (23.5)
Fetal position		
OA	14 (73.7)	14 (82.4)
OT	2 (10.5)	2 (11.8)
OP	3 (15.8)	1 (5.9)
Fetal head palpable per abdomen		
0/5	19 (100.0)	17 (100.0)
1/5	0 (0.0)	0 (0.0)
Station of fetal head		
Spines	0 (0.0)	3 (17.6)
+1 cm below the spines	6 (31.6)	10 (58.8)
+2 cm below the spines	12 (63.2)	4 (23.5)
+3 cm below the spines	1 (5.3)	0 (0.0)
Molding		
None	0 (0.0)	0 (0.0)
+	8 (42.1)	1 (5.9)
++	9 (47.4)	13 (76.0)
+++	1 (5.3)	3 (17.6)
Caput		
None	1 (5.3)	0 (0.0)
1 cm	8 (42.1)	1 (5.9)
2 cm	3 (15.8)	6 (35.3)
3 cm	7 (36.8)	7 (41.2)
Analgesia^c^		
None	1 (5.3)	0 (0.0)
Perineal infiltration	7 (36.8)	2 (11.8)
Pudendal block	0 (0.0)	0 (0.0)
Regional anesthesia	15 (78.9)	15 (88.2)
General anesthesia	0 (0.0)	0 (0.0)
Grade of operator		
Attending	14 (73.7)	13 (76.5)
Trainee	5 (26.3)	4 (23.5)

Data are presented as mean±standard deviation, number (percentage) or median (interquartile range).

*BMI*, body mass index; *OA*, occiput anterior; *OP*, occiput posterior; *OT*, occiput transverse.

*Hotton et al. Novel Odon Device and operative vaginal birth. Am J Obstet Gynecol 2021.*

a n=16

b n=15

c more than 1 type of analgesia can be used.

### Clinical birth outcomes

#### Neonatal outcomes

Table 3 summarizes neonatal outcomes. In this study, 2 infants (1 successful Odon Device and 1 failed Odon Device followed by nonrotational forceps, both cases assisted for “presumed fetal compromise”) had an Apgar score of <7 at 5 minutes of life (both Apgar scores of 6). No infant was born with an Apgar score of <7 at 5 minutes where the indication to assisted birth was prolonged second stage of labor. In addition, 3 infants (8%) were admitted to the neonatal intensive care unit (NICU) following birth, all with respiratory distress. In all 3 cases, the indication for operative vaginal birth was “presumed fetal compromise.” NIPS was reassuring in only 1 infant, who was admitted to the NICU, with a score of ≥4 indicating pain.

**Table 3:** Immediate neonatal outcomes

Neonatal outcome	Overall (n=40)	Successful Odon (n=19)	Unsuccessful Odon (n=21)
Umbilical artery pH	7.18±0.07	7.19±0.06^a^	7.18±0.08^b^
Umbilical artery base excess	−6.6 (−8.6 to −5.2)	−6.5 (−8.5 to −5.7)^a^	−7.0 (−9.0 to −4.6)^b^
Umbilical vein pH	7.33 (7.29 to 7.36)	7.33 (7.30 to 7.36)^c^	7.31 (7.28 to 7.36)^b^
Umbilical vein base excess	−4.9 (2.3)	−4.9 (2.4)^c^	−4.8 (2.3)^b^
Shoulder dystocia	1 (2.5)	0 (0.0)	1 (4.8)
Apgar scores<7			
1 min	5 (12.5)	3 (15.8)	2 (9.5)
5 min	2 (5.0)	1 (5.3)	1 (4.8)
10 min	0 (0.0)	0 (0.0)	0 (0.0)
Neonatal Infant Pain Scores≥4			
2 h postnatal^b^	1 (2.6)^d^	1 (5.6)^e^	0 (0.0)
6 h postnatal^c^	0 (0.0)	0 (0.0)	0 (0.0)

Data are presented as mean±standard deviation, number (percentage) or median (interquartile range).

*Hotton et al. Novel Odon Device and operative vaginal birth. Am J Obstet Gynecol 2021.*

a n=14

b n=19

c n=17

d n=39

e n=18.

Most neonatal events (16 of 17) were attributed to soft tissue trauma (ie, bruise, graze, scalp injury, or facial injury) (Table 4). There was less soft tissue trauma in infants successfully delivered with the use of the Odon Device; 3 babies (16%) whose birth was successfully assisted with the Odon Device had evidence of soft tissue trauma compared with 12 cases (60%) in which the device failed. In addition, 4 infants (10%) had a cephalohematoma diagnosed at their postnatal checkup (2 successful and 2 unsuccessful cases). No infant required phototherapy for jaundice contributed to by bruising, received a blood transfusion, had a neonatal seizure, was therapeutically cooled, was diagnosed with organ failure, or died within 28 days following birth.

**Table 4:** Neonatal outcomes up to day 28

Variable	Overall (n=40)	Successful Odon (n=19)	Unsuccessful Odon (n=21)
Admitted to the NICU at any point up to day 28	3 (7.5)	2 (10.5)	1 (5.0)
Neonatal events diagnosed or still present between NIPE and day 28			
Any neonatal event	17 (43.6)	4 (21.1)	13 (65.0)
Neonatal soft tissue trauma	15 (38.5)	3 (15.8)	12 (60.0)
Pressure necrosis of skin or fat	0 (0.0)	0 (0.0)	0 (0.0)
Cephalohematoma	4 (10.3)	2 (10.5)	2 (10.0)
Neonatal vascular injury	0 (0.0)	0 (0.0)	0 (0.0)
Neonatal skeletal injury	0 (0.0)	0 (0.0)	0 (0.0)
Other	1 (2.6)	1 (5.3)	0 (0.0)

*NICU*, Neonatal intensive care unit; *NIPE,* newborn and infant physical examination.

*Hotton et al. Novel Odon Device and operative vaginal birth. Am J Obstet Gynecol 2021.*

Furthermore, 11 infants (28%) experienced a serious adverse event (SAE) defined as an event that required hospitalization or prolongation of hospital stay or further intervention: 3 cases of jaundice requiring phototherapy not contributed to by bruising; 2 cases of respiratory difficulties requiring NICU admission; 1 prolonged neonatal stay for intravenous antibiotics secondary to maternal sepsis in labor; 1 hospitalization for weight loss; and 1 readmission for bronchiolitis aged 10 days. None of the cases were directly attributable to the use of the Odon Device.

#### Maternal complications and clinical features

Table 5 summarizes maternal outcomes. In this study, 36 of 40 women (90%) had a perineal tear (28 episiotomies). The rate of episiotomy in successful Odon Device–assisted births was the same as when forceps were used—14 of 19 cases (74%), respectively. Furthermore, 3 women (8%) sustained a third-degree perineal tear: 1 (3%) during the successful use of the Odon Device and 2 (5%) when forceps were used following a failed Odon attempt. In addition, 1 cervical tear (3%) (which did not require suturing) was identified after a successful Odon Device–assisted delivery.

**Table 5:** Maternal outcomes

Variable	Overall (n=40)	Successful Odon (n=19)	Unsuccessful Odon (n=21)
Weighed blood loss (mL)	499 (355–810)	514 (420–746)	450 (300–1302)
Perineal tears			
None	4 (10.0)	1 (5.3)	3 (14.3)
First degree	3 (7.5)	1 (5.3)	2 (9.5)
Second degree	7 (17.5)	2 (10.5)	5 (23.8)
Episiotomy	28 (70.0)	14 (73.7)	14 (66.7)
3A	1 (2.5)	0 (0.0)	1 (4.8)
3B	2 (5.0)	1 (5.3)	1 (4.8)
3C	0 (0.0)	0 (0.0)	0 (0.0)
Fourth degree	0 (0.0)	0 (0.0)	0 (0.0)
Defect in the ischiorectal fossa	2 (5.0)	1 (5.3)	1 (4.8)
Cervical tear present	1 (2.5)	1 (5.3)	0 (0.0)
Cervical tear requiring suturing	0 (0.0)	0 (0.0)	0 (0.0)
Labial tear requiring suturing	1 (2.5)	1 (5.3)	0 (0.0)
Postnatal perception of pain			
Day 1	4 (3.0–6.0)	4 (3.0–6.0)	4 (3.0–6.0)
Day 7	3 (1.5–4.0)	3 (1.0–5.0)	3 (2.0–4.0)
Day 28	1 (1.0–2.0)	1 (1.0–1.0)	1 (1.0–2.0)
Symptoms of fecal or flatal incontinence at day 90			
Never	0 (0.0)^a^	0 (0.0)^b^	0 (0.0)^b^
Rarely	32 (88.9)^a^	16 (88.9)^b^	16 (88.9)^b^
Sometimes	1 (2.8)^a^	0 (0.0)^b^	1 (5.6)^b^
Often	3 (8.3)^a^	2 (11.1)^b^	1 (5.6)^b^
Always	0 (0.0)^a^	0 (0.0)^b^	0 (0.0)^b^
Symptoms of urinary incontinence at day 90	13 (36.0)^a^	5 (27.8)^b^	8 (44.4)^b^

Data are presented as number (percentage) or median (interquartile range).

*Hotton et al. Novel Odon Device and operative vaginal birth. Am J Obstet Gynecol 2021.*

a n=36

b n=18.

The median weighed blood loss was 499 mL (25th 355 mL, 75th 810 mL). Furthermore, 2 women (5%) received a postnatal red blood cell transfusion. In both cases, the Odon Device had been unsuccessful, and nonrotational forceps were used, resulting in hemorrhage from perineal trauma.

In addition, 7 women (18%) experienced an SAE: 3 third-degree tears, 2 postpartum hemorrhages required blood transfusion, 1 postnatal infection, 1 complication from a known neurologic condition, and 1 case of fecal incontinence at 28 days after birth.

Women reported a better health score (standardized EQ-5D-5L health-related quality-of-life questionnaire) at day 28 (mean, 88.1; standard deviation [SD], 8.6) compared with antenatally (mean, 82.4; SD, 12.0) and on day 1 postnatal (mean, 77.1; SD, 16.1).

#### Device outcomes

In the 19 cases in which the Odon Device assisted the birth of the baby, the median time between the application of the device and time of birth was 5 minutes (25th 4, 75th 7), with a median “decision-to-delivery” interval of 11 minutes (25th 9, 75th 17). There were 21 births where the Odon Device did not assisted the birth of the baby: a manufacturing error affecting 4 devices accounted for 19% of the failed births; 3 failures (14%) occurred when the vertex was at the level of the ischial spines; further failures were attributed to (1) failure to reach “0” during the application of the device (n=6, 29%), (2) operator learning curve (n=3, 14%), and (3) no reason identified (n=5, 24%).

The operator reported that the device was “easy” or “very easy” to “set up” and “deflate the air chamber” in 38 (95%) and 35 (88%) of 40 cases, respectively. The application of the device and the withdrawal of the applicator were more challenging: only 21 (53%) and 20 (50%) cases were reported as “easy” or “very easy,” respectively.

**Figure 5:**
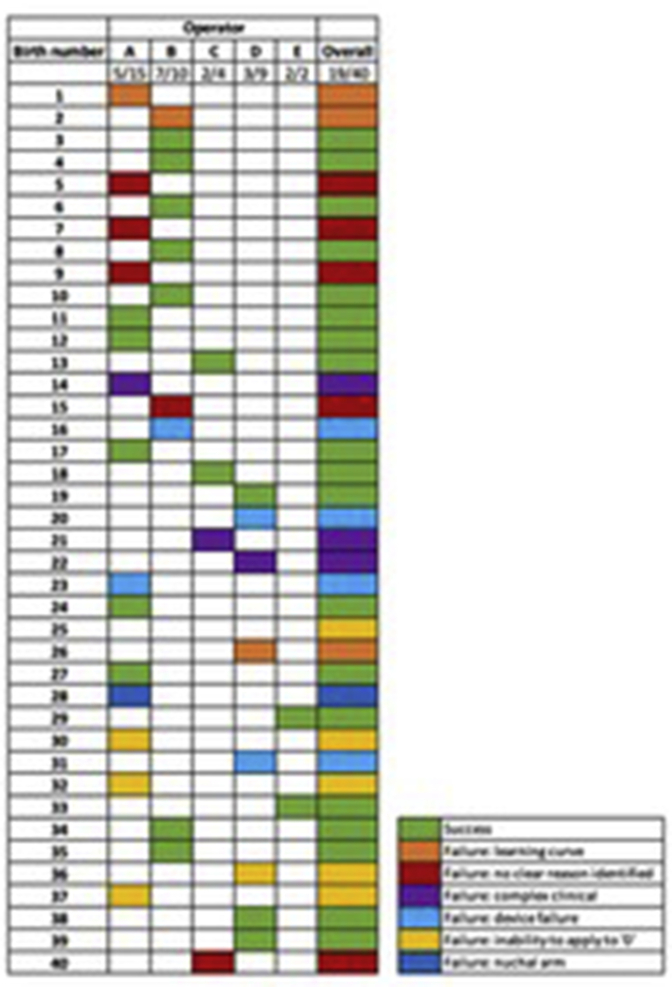
Details of device failure and success by category and operator *Hotton et al. Novel Odon Device and operative vaginal birth. Am J Obstet Gynecol 2021.*

The 5 operators joined the study in stages. The number of births and success rates per operator were variable (Figure 5). Operator A was successful in 5 of 15 births (33%), operator B in 7 of 10 births (70%), operator C in 2 of 4 births (50%), operator D in 3 of 9 births (33%), and operator E in 2 of 2 births (100%).

## Comment

### Principal findings

It is feasible to recruit women to a study investigating a novel device for operative vaginal birth. A recruitment rate of 78% is high and appears to be related to a general maternal aspiration for an alternative to forceps and vacuum (details from qualitative findings that will be published separately). The success rate of the Odon Device was lower than the reported success rates of both vacuum and forceps^12^^,^^19^; however, the device had been used to induce birth where clinically indicated.

### Results

Factors that are likely to affect the efficacy of the device assisted the clinical context, technique of application and traction, the initial uncertainty of the optimal technique in inserting and using the device (as the device was used for clinically indicated cases), and the design and functionality of the device itself. For example, it became clear after 3 births that the device is most effectively placed during a uterine contraction, in contrast to forceps and vacuum.

Although the study was not powered to look at predictors, births were more likely to be successful if the fetal vertex was lower in the birth canal or if there was minimal molding of the fetal skull or similar other assisted births.^12^ The Odon Device was never successful when it was used with the vertex at the level of the ischial spines; indeed, a vertex above +1 is a contraindication to attempting operative vaginal birth in some national guidelines.^20^ The average time of decision to delivery was 11 minutes, which was faster than documented average times in the literature of 15 to 59 minutes depending on whether the birth was assisted in a labor or operating room.^21^, ^22^, ^23^ The Odon Device was successfully employed across the full range of fetal positions using the same technique, which may be particularly useful for maternity settings with low operative vaginal birth rates. During the routine device inspection following birth, 4 devices (10%) were found to have a fault with the inflation mechanism. Further investigation along with the manufacturer identified a single issue related to the use of gamma radiation in the sterilization process that degraded the device components. This issue has been rectified by the manufactures and highlights the importance of postuse device inspection during feasibility studies.

### Clinical implications

There was no significant maternal or neonatal safety concern associated with the use of the device, although the number of births is small. There seem to be some neonatal benefits associated with the use of the device, consistent with the findings from simulation^11^; 16% of babies whose birth was successfully assisted with the Odon Device had evidence of soft tissue trauma compared with 60% of babies in cases where the device was unsuccessful. We recorded any soft tissue trauma, including a bruise, graze, laceration, or hematoma, whether it appeared to be related to device use or not. Evidence demonstrated that the laceration rate following operative vaginal delivery can be up to 10%^22^ and instrument bruises up to 37%^24^; however, there are few studies reporting all soft tissue injury. We had 4 cephalohematomas (10.3%), which agrees published rates of occurrence following operative vaginal birth.^22^ Clearly, the use of 2 instruments is associated with an increased risk of neonatal trauma,^25^ but the trauma rates in this study were consistent with other operative vaginal birth studies in the same unit.^26^ Operators used their clinical judgment to decide whether to use a second device or perform a cesarean delivery following the failure of a primary device. This study routinely used NIPS to assess infants following operative vaginal birth, and the scores have indicated reassuringly low levels of neonatal pain.

Maternal outcomes were also acceptable. No woman required a blood transfusion after a successful Odon Device–assisted birth compared with 10% of women in unsuccessful cases. Clearly, this may reflect the use of 2 instruments^25^ and/or a more difficult cohort of births. Overall, the maternal outcomes were again comparable to a previous study of operative vaginal birth in the study unit.^26^ There were 3 third-degree tears (7.5%) during the study, 1 (5.3%) from a birth successfully assisted by the Odon Device and 2 (9.5%) from births requiring a second device to assist birth. This agrees tear rates in the literature, which range from 1% to 4% for ventouse-assisted births,^22^ 8% to 12% for forceps-assisted births,^22^ and 17.4% for women who had births assisted with sequential devices.^25^^,^^27^

This study routinely collected maternal birth perceptions as part of the assessment of operative vaginal birth. It is encouraging that 98% of women in the study rated their birth very positively.

### Research implications

A future randomized controlled trial will be required to objectively compare outcomes among the Odon Device, forceps, and vacuum. However, this would require equipoise among the options for operative vaginal birth. We are confident that the success rates of the Odon Device can be improved with iterative improvements to the insertion technique, refinement of participant selection, and a more reliable device design. Further feasibility studies are currently underway in Bristol, United Kingdom (https://www.nbt.nhs.uk/research-innovation/our-research/current-research/women-childrens-health-research-unit/women-4), and Besançon, France, and the success rate has markedly improved in this second phase; an interim analysis of the current ongoing studies demonstrated a success rate of 78% (66 of 85 cases).^28^

### Strengths and limitations

The Odon Device has been used in clinically indicated cases for operative vaginal birth. It demonstrates an innovative approach to information sharing and recruitment, with positive results. The study ensured a holistic approach when evaluating a novel device, ensuring that data from women, infants, clinicians, midwives, and the device were scrutinized. Key quantitative data were consolidated with embedded qualitative research to enable further understanding. Key limitations included the small sample size and the reasonably short follow-up period of 90 days. We intended to perform long-term follow-up of women and their babies during the next phase of the study, following the completion of our feasibility research. We acknowledge that this research was undertaken in a single center in a single country where most participants were White.

### Conclusions

Investigating a new device for operative vaginal birth is both feasible and supported by women. The potential advantages of the Odon Device (eg, possible reduction in neonatal soft tissue trauma, single mode of application irrespective of fetal position) merit further iterative exploration and investigation of the device in larger studies.

### Highlights

• The Odon Device may offer women an alternative instrumental birth.
• There was no maternal or neonatal safety concerns in the births.
• Recruitment rate was higher than expected.

## Figures and Tables

**Supplemental Table 1:** Patient Perception Score

Women’s perception of birth						
Question	Strongly disagree	Disagree	Neutral	Agree	Strongly agree	Score
I felt well informed due to good communication	1	2	3	4	5	
I felt I was treated with respect at all times	1	2	3	4	5	
I felt safe at all times	1	2	3	4	5	
						Total

*Hotton et al. Novel Odon Device and operative vaginal birth. Am J Obstet Gynecol 2021*.

**Supplemental Table 2:** Practitioner-reported outcome

Practitioner-reported outcomes					
Perceived overall ease of use of device	Very difficult				Very easy
	1	2	3	4	5
Ease of instrument setup	Very difficult				Very easy
	1	2	3	4	5
Ease of instrument application to the baby’s head	Very difficult				Very easy
	1	2	3	4	5
Ease of withdrawal of applicator after application	Very difficult				Very easy
	1	2	3	4	5
Comfort with the level of force required to deliver the baby	Very difficult				Veryeasy
	1	2	3	4	5
Ease of deflation of the air chamber prior to crowning	Very difficult				Very easy
	1	2	3	4	5
Any other comments or impressions regarding the delivery with the Odon Device?	Please insert free comments below:				

*Hotton et al. Novel Odon Device and operative vaginal birth. Am J Obstet Gynecol 2021*.

## Supplementary Data

Video Demonstration of the use of the Odon Device *Hotton et al. Novel Odon Device and operative vaginal birth. Am J Obstet Gynecol 2020.*
